# Growth of the Black Soldier Fly *Hermetia illucens* (Diptera: Stratiomyidae) on Organic-Waste Residues and Its Application as Supplementary Diet for Nile Tilapia *Oreochromis niloticus* (Perciformes: Cichlidae)

**DOI:** 10.3390/insects13040326

**Published:** 2022-03-25

**Authors:** Rafael Pérez-Pacheco, Demián Hinojosa-Garro, Fernando Ruíz-Ortíz, Juan Carlos Camacho-Chab, Benjamín Otto Ortega-Morales, Nancy Alonso-Hernández, Alicia Fonseca-Muñoz, Nadia Landero-Valenzuela, Henry Jesús Loeza-Concha, Fidel Diego-Nava, Fabián Arroyo-Balán, Carlos Alejandro Granados-Echegoyen

**Affiliations:** 1 Centro Interdisciplinario de Investigación para el Desarrollo Integral Regional (CIIDIR), Instituto Politécnico Nacional, Oaxaca, Calle Hornos 1003, Colonia Noche Buena, Oaxaca 71230, Mexico; rafaelperezpacheco@yahoo.com (R.P.-P.); 91fernando.iz@gmail.com (F.R.-O.); alonsoh_nancy@hotmail.com (N.A.-H.); fdiego@ipn.mx (F.D.-N.); 2 Laboratorio de Ecología Acuática, Centro de Estudios en Desarrollo Sustentable y Aprovechamiento de la Vida Silvestre (CEDESU), Universidad Autónoma de Campeche, Avenida Héroe de Nacozari #480, Campeche 24079, Mexico; dhinojos@uacam.mx; 3 Departamento de Microbiología Ambiental y Biotecnología (DEMAB), Universidad Autónoma de Campeche, Av. Agustín Melgar, Colonia Buenavista, Campeche 24039, Mexico; juanccam@uacam.mx (J.C.C.-C.); beortega@uacam.mx (B.O.O.-M.); 4 Escuela de Sistemas Biológicos e Innovación Tecnológica, Universidad Autónoma “Benito Juárez” de Oaxaca (SBIT-UABJO), Av. Universidad s/n, Ex-Hacienda 5-Señores, Oaxaca 68120, Mexico; afonsem@gmail.com; 5 Departamento de Biociencias y Agrotecnología, Centro de Investigación en Química Aplicada, Coahuila 25294, Mexico; nadialv@hotmail.com; 6 Colegio de Postgraduados Campus Campeche, Carretera Haltunchén-Edzná km 17.5, Sihochac, Campeche 24450, Mexico; loeza.jesus@colpos.mx; 7 Laboratorio de Entomología Aplicada, Centro de Estudios en Desarrollo Sustentable y Aprovechamiento de la Vida Silvestre (CEDESU), CONACYT-Universidad Autónoma de Campeche, Avenida Héroe de Nacozari 480, Campeche 24079, Mexico; fabian.arroyo@conacyt.mx

**Keywords:** organic urban waste, fish-meal, economic alternative, *Hermetia*

## Abstract

**Simple Summary:**

The need to identify sustainable and profitable resources for the elaboration of useful feeds in animal nutrition has been the focus of many studies. Insect-based feeds have been tested by introduction into the diets of pigs, poultry, and fish, with interesting results. Worldwide, solid waste management has increased because of economic and population growth, and most investigations have concentrated on identifying efficient solutions to reduce this waste. Aquaculture has experienced exceptional growth and is perceived as having the greatest potential to meet the growing demand for food in the world, by using aquatic species that serve as a protein source in the diet and therefore contribute to the food security of the population. There are studies with the use of black soldier flies (BSF) as a potential feed agent for animals with high protein content. The relevance of evaluating different food resources in the BSF-immature stages is important because the quantity and quality of the food provided to the fly larvae generate different responses on the growth and development of this insect used as animal feed.

**Abstract:**

The black soldier fly, *Hermetia illucens* (BSF, Diptera: Stratiomyidae) is an insect with high protein value and a potential feed agent for animals aimed for human consumption. The growth parameters of BSF larvae reared on four substrates—restaurant-waste, fruit-waste, fish-waste, and commercial tilapia food—for 41 days before processing for inclusion into *Oreochromis niloticus* (Perciformes: Cichlidae, Nile tilapia) commercial fry diets at 30% (70:30) were determined. On fly larvae, the food substrate based on restaurant waste yielded the greatest larval weight and length. BSF larvae fed a fish-waste diet showed the shortest developmental time. The fruit-waste diet induced the lowest weight and length in the fly larvae/pre-pupae (immature stage). The pre-pupal protein values were similar to commercial food. On fry-fish, the diets with pre-pupae grown on fish waste showed the greatest yields regarding weight (biomass), length, and nutritional content. These results suggest the BSF has the potential to be used in fish feed and provides an alternative for commercial cultivation.

## 1. Introduction

Worldwide, solid waste management has increased because of economic and population growth [1,2]. It is common to dispose of this waste in streets or open dumps, blocking drains, attracting disease vectors, and causing public health problems [3]. Recently, increasing research has been focused on developing efficient solutions to degrade and re-use these resources. One of them is using waste as a food substrate for insect populations, which can serve as a protein source for the production of animal feed [4]. Many studies have focused on the need to identify sustainable and profitable resources as functional foods in animal nutrition. There were interesting results when insect flour was incorporated as a protein source into diets for pigs, poultry, and fish [1,5,6,7,8,9,10,11,12]. Among the insects used in aquaculture feeding, arthropods belonging to the taxonomic orders Orthoptera, Coleoptera, Isoptera, Lepidoptera, and Diptera have been cited [7]. The Diptera order is one of the largest groups of insects, with approximately 100 families and 85,000 described species, most of which have economic importance because they participate in organic-matter degradation [13]. The Stratiomyidae family includes the Brachycera sub-order with 2600 species registered worldwide [14,15,16].

The black soldier fly *Hermetia illucens* L. (BSF, Diptera: Stratiomyidae) is distributed in the tropics and is found in humid and nutrient-rich ecosystems with an abundance of decomposing animal and plant remains; larval nutritional composition can reach up to 40% protein and 30% fat, and varies depending on the substrate used for growth [17,18]. There are also records of growth improvement in poultry, pigs, and commercial fish species fed with insect flour [8]. Protein and fat values observed in these flours meet the requirements established for fish feeding by the Organization of the United Nations for Food and Agriculture (FAO) [19].

BSF larvae have a significant protein content and possess a well-balanced amino acids and fats. Nutritional composition and fatty acid profiles are dependent on the developmental stage [20], making them a good nutritional source of animal feed. BSF is an excellent organic-waste bioconversion organism, but the type of substrate where BSF is reared is quite important for improved bioconversion activity, because of the nutrients needed to support its growth [21,22,23]. Several studies concentrate their aim on BSF larval nutrient assimilation in response to specific rearing conditions and substrates to improve their yield and quality [24,25]. In addition, BSF-derived products such as fats can be used as biodiesel [20], and oil can be used for medical proposes and as a skincare product [26]. Another valuable product gained from BSF is chitin—chitosan is widely used in medicine, cosmetics, and biotechnological research [27]. Hence the importance of black soldier fly research.

Several substrates such as manure, butchery, and vegetable wastes [28], fruits [29], kitchen waste [30], and coffee by-products (coffee silverskin) enriched with 10% of *Schizochytrium* sp. have been tested for BSF larval growth [31]. Each substrate confers different conditions for larvae growth and different nutrition values, development time, size, and biomass [32]. For instance, Scala et al. [33] found that larvae fed on diets containing spent grain contained 15% or more protein than those larvae reared only on fruit

Aquaculture has experienced exceptional growth and is perceived as having the greatest potential to meet the growing demand for food worldwide by using aquatic species that serve as a protein source in the diet and therefore contributing to the food security of the population [34,35]. In Mexico, exploitation of Nile tilapia (*Oreochromis niloticus* L., Perciformes: Cichlidae) is an option that has been promoted to generate income for inhabitants of tropical areas. This has been accepted because of its ease of cultivation. However, rural producers in the southeast of the country face a constant problem with the cost, scarcity, and quality of commercial balanced foods [36]. The importance of evaluating different food sources for the BSF resides in the fact that factors such as moisture, nutritional composition, quantity, and quality of the food provided to the larvae may generate different responses in the development and growth of this insect and can also affect survival in larval stages and nutrient assimilation [28,37].

The present study investigated the effects on *O. niloticus* growth performance of partial dietary replacement of fishmeal by BSF prepupae cultured on four different organic substrates. A BSF stock colony was also set up to determine BSF survival and the development time for several stages, thus identifying parameters that would benefit the establishment of an optimized production breeding program (weight/biomass, length, and nutritional content). We hypothesized that larval development and biomass gain of fish depends on the nutrient content of the type of substrate on which the larvae grow.

## 2. Materials and Methods

### 2.1. Insect Rearing

The flies were reared in the facilities of the Applied Entomology Laboratory (LEA as in its Spanish abbreviation) of the Benito Juárez Autonomous University of Oaxaca (UABJO as in its Spanish abbreviation, 17°03′17.6″ N, 96°43′37.6″ W) in a 4 × 3 × 3 m concrete room with an artificial lighting system (Master-TL5 High Output Eco 109 LM/W Philips Lighting Holding B.V.), 12:12 light and dark cycle under a constant temperature of 27.00 ± 1.03 °C, and relative humidity of 51.77 ± 6.23% [38]. Eggs were obtained from a colony of flies established in the LEA-UABJO and reared in a 100 × 80 × 80 cm entomological cage where the larvae fed ad libitum. A mixture of 500 g of commercial tilapia food (Atilapia^®^, Aquaplus-Campi, Mexico) with 800 mL of distilled water in a plastic container (5 L) was prepared, then corrugated cardboard strips (to encourage oviposition into the holes of the cartons) on the food mixture were placed inside of the brooding cage to collect the eggs [39].

### 2.2. Substrates for Fly-Larvae Development

The larvae were developed on four different substrates: (1) restaurant-waste composed (approximately) of cooked chicken meat (5%), pork (5%), beef (5%), cooked tomato (20%), corn tortilla (50%), and rice (15%); (2) fruit-waste composed of cucumber (10%), pineapple (20%), watermelon (20%), mango (25%), papaya (20%), and carrot (5%) residues; (3) fish-waste composed of muscle (40%), skin (25%), and gills (35%) of *Solea solea* L. (Pleuronectiformes: Soleidae); and (4) commercial tilapia food (Atilapia^®^) as a control (40.13% crude protein, 5.96% crude fat, 12.00% moisture, and 10.03% ash) [37]. The chemical-nutritional characteristic of each substrate was performance.

### 2.3. Black Soldier Fly Growth

A cardboard strip with packets of BSF eggs (*n* = 150) was placed on 40 g of each food substrate in a 2 L plastic container. Three days after hatching, we recorded data every 24 h until 40% of the larvae had reached the pre-pupal stage and until the insects reached the adult phase [39]. Three replicates per food substrate were carried out. Ten (10) larvae were sampled in each replica and then returned to their respective container [37,38]. The length and gained weight for each larva were measured with a Vernier caliper (Model-CVQ1315) and an electronic scale, respectively (Scout^®^ Pro, Ohaus Corporation, Germany).

### 2.4. Nile Tilapia Growth Test

For fish-diet preparation, pre-pupae reared on the mentioned food substrates were used. Pre-pupae were dehydrated in an oven at 90 °C (Felisa EU) for 96 h and then crushed using a mechanical mill and the obtained powder mixed with grounded commercial tilapia food (Atilapia^®^) at a ratio of 30% [40,41,42]. The resulting mixture was moistened with distilled water, obtaining a slurry with a semi-liquid consistency which then was mixed in a blender for 10 min. Flat surface metal trays were prepared, and a thin layer (0.2 mm) of the slurry was spread and allowed to dry at room temperature for 48 h. The flakes were removed with the help of a spatula and packed into flexible polyethylene zip-lock bags.

For this bioassay, hormonal 7-day-old *O. niloticus* fries were used. The fingerlings were obtained from Laboratorio Central Acuícola S.A., in the State of Campeche, Mexico (19°45′33.41″ N, 90°28′00.59″ W). The fish fry were acclimatized for two days in a 1000 L tank at the facilities of the Aquatic Ecology and Environmental Monitoring Laboratory of the Center for Studies in Sustainable Development and Use of Wildlife (CEDESU), at the Autonomous University of Campeche (UAC) (19°48′07.63″ N, 90°30′16.31″ W). For the experiment, 12 aquaria, with a capacity of 38 L each, were used. The aquaria were oxygenated using air pumps (Elite model A-802) and the oxygen concentration kept between 5.0 and 9.0 mg/L. The seeding density was 3.75 L/fish.

The fish were fed with 30% BSF-pre-pupae flour inclusion in commercial food with 100% commercial tilapia food (Atilapia^®^) as a control group. The fish-fry feed was carried out by supplying 5% of the total weight of the fish biomass at four-hour intervals, starting at 08:00 each day [43]. The amount of food was provided according to the increase in average weight and biomass of each sampling. The fish fry were randomly distributed in each aquarium, and each diet experiment was carried out in triplicate (*n* = 3). Sampling was carried out every six days, with total length and biomass gain (wet weight) of fish recorded with an ichthyometer (model KH-PISCIS-60-22) and an electronic scale (Scout^®^ Pro, Ohaus Corporation, ±0.001 g sensitivity). The salinity, dissolved oxygen, and water column temperature were recorded daily using a portable probe (HACH model HQ40d).

### 2.5. Nutritional Analysis of the BSF-Diet and Muscle of Nile Tilapia

For this, 100 g of the wet matter was used and placed on an aluminum tray. The samples were dried in a convection oven (PerfectBroil, CTO4400B, Black & Decker Corporation, Towson, MA, USA) at 100 °C for 15 min and cooled before recording dry weight. The samples were then placed in a mill (Fritsch Pulverisette-14), pulverized, and stored in zip-lock polyethylene bags. For moisture determination, 2 g of the ground matter was weighed and placed in the oven at 105 ± 0.05 °C for 24 h until a constant weight was achieved [44,45]. Moisture was calculated using the following formula:

(a) Moisture (%) = ((wet weight-dry weight)/(wet weight)) × 100

(b) Dry matter (%) = 100 − Humidity (%)

### 2.6. Ash Determination

For this, 2 g of ground matter were weighed from each treatment and placed into a 50 mL crucible. These samples were then placed into a Felisa^®^ MM 60 muffle at 500 °C for one hour to obtain carbon-free ash. Ash percentage was determined by the following formulae [46]:

(a) Wet base ash = ((ash weight + crucible weight) − (crucible weight × 100))/(weight of the sample)

(b) Dry base ash = ((wet base ash (%))/(100 − moisture (%))) × 100

### 2.7. Determination of Crude Fat Content

The crude fat content was determined according to the Mexican standard and AOAC [44] procedures by using the ethereal extract determination (Soxhlet) method for meat products. For this, 2 g of the sample was weighed, placed on filter paper, covered with a cotton portion, and placed into the extractor. A 1000 mL flat-bottomed balloon flask was placed in the lower area, and 80 mL of ether was added to the upper end of the condenser. The extraction was carried out for six hours. The ether in the flask was evaporated and dried to constant weight. Fat content was quantified by weight difference using the following equation:

Fat (%) = ((PG − P)/m) × 100

where PG is the mass in grams of the flask with fat, P is the mass in grams of the empty flask, and m is the mass in grams of the sample.

### 2.8. Determination of Proteins in the Diet Based on Pre-Pupae

A fresh sample of diets based on pre-pupae was used with bovine serum albumin as a standard at 1 mg/mL. Each sample was homogenized with a mortar. For this, 1 g of protein was measured with a digital weighing scale and mixed with 9 mL of sucrose solution. This mixture was centrifuged at 4000 rpm for 8 min. Then the supernatant was collected and 1 mL of protein sample was added to 4 mL of alkaline copper solution using a vortex shaker. These solutions were incubated at room temperature for 12 min. After this, 0.5 mL of Folin–Ciocalteau phenol was added to these solutions and mixed by using a vortex shaker. Finally, these solutions were incubated for 30 min at room temperature and in the dark. All absorbance readings were carried out in triplicate at 750 nm using a UV–VIS spectrophotometer (Thermo Scientific, Waltham, MA, USA) [47].

### 2.9. Determination of Proteins in the Muscle of Nile Tilapia

For this, 0.25 g of fish-muscle tissue samples from Nile tilapia was weighed and placed into an 800 mL balloon flask. The sample (6 mL) was digested in an oven at 400 °C with concentrated sulfuric acid in the presence of potassium sulfate and a catalyst (half a Kjeldahl tablet: K_2_SO_4_, 48.40%; Na_2_SO_4_, 48.30%; CuSO_4_, 0.30%) to speed up the digestion and obtain nonvolatile ammonium sulfate. After cooling, 30 mL of distilled water was added, and the ammonium sulfate was converted to volatile ammonia gas by heating with 20 mL of sodium hydroxide. Then 10 mL of boric acid solution was added to the steam-distilled ammonia, which was trapped by forming ammonium borate. The amount of borate formed was determined by titration using hydrochloric acid [44]. The protein content was calculated using the following formulae:

(a) Nitrogen (%) = ((g nitrogen in sample)/(g of sample)) × 100

(b) Protein (%) = % N (total nitrogen content) × Kjeldahl factor for meats (6.25)

### 2.10. Ethical Standards

Our experiments were carried out according to the ethical procedures for the production, care, and use of laboratory animals of the Mexican National Standard [48].

### 2.11. Experimental Design and Statistical Analysis

All experiments were carried out using a completely randomized design with three replicates and data presented as the mean ± standard deviation (SD) to provide an idea of the magnitude of the differences between means. The data were tested to verify the normality of errors (Shapiro–Wilk test) and homogeneity of variances (Bartlett test). One-way analysis of variance (ANOVA) was performed and mean comparison using the Tukey test (*p* < 0.05) as a post-hoc test carried out with the aid of Minitab version 18.1 to evaluate egg-hatching time, larvae–pupae developmental days, percentage of larval survival, larval–prepupal weight (g), and length (mm) for each substrate tested (restaurant waste, fruit waste, fish waste, and commercial food), along with the total caudal length (cm) and weight gain (biomass) of fish development for each diet containing 30% of larvae grown in each substrate and 100% commercial food as a control. In the study, these data were included as factors in the analysis. The nutritional composition percentages (moisture, ashes, protein, and fat) of BSF pre-pupae meal and muscle of *O. niloticus* fed with the diets were also evaluated.

## 3. Results

### 3.1. Survival and Development of BSFs

Significant differences in BSF development were found (Table 1). The restaurant-waste-based substrate extended the biological cycle of the BSFs to 40.50 ± 0.50 days (d) when compared to the rest of the food substrates, whereas the control group registered a cycle of 33.00 ± 0.81 d. BSF fed on fruit waste registered a development rate of 34.87 ± 0.85 d from egg to adult emergence. The fish-waste-based substrate induced a lower egg–adult development period than the rest of the treatments (31.33 ± 0.47 d). We suggest that the larvae could complete their cycle in a shorter period because of the amount of protein and fat provided by this waste. The control substrate showed the highest larval survival percentage at 98.88 ± 1.92%, contrary to that recorded by restaurant-waste treatment, which yielded the lowest rate (76.66 ± 3.33%) (F_1,3_ = 8,23; *p <* 0.008; r^2^ = 75.53). The fish-waste treatment registered a larval survival of 88.00 ± 1.15%. Table 2 shows the chemical composition of the substrates used in the trial.

### 3.2. Weight (Biomass) and Length of Fly Larvae/Pupae

Substrate significantly affected the total weight of pre-pupae. Pre-pupae reared on restaurant waste were higher in biomass (29.9 g on average considering the total number of larvae and larval survival) registering a weight-gain of 0.25 ± 0.04 g, which represents an increase of 16 to 32% when compared to fruit and fish waste (19.59 and 27.72 g on average) (F_3,11_ = 12.90; *p <* 0.001; r^2^ = 25.01). The fruit-waste substrate produced a significantly lower weight (0.17 ± 0.04 g) when compared to the rest of the treatments (F_3,11_ = 29.00; *p <* 0.001; r^2^ = 42.86). There were significant differences between pairs of means from restaurant-waste and control groups vs. fruit and fish-waste groups. The first two treatments produced the highest weight (23.78 ± 1.86 g and 23.19 ± 2.61 g, respectively). BSF in all substrates showed a constant increment before harvesting. The larval length registered for restaurant-waste treatment was significantly lower (23.59 ± 1.84 mm) compared to the rest of the treatments (<12.2–19.45%) (F_3,11_ = 18.06; *p <* 0.001; r^2^ = 31.84) (Table 3).

### 3.3. Bromatological Composition of the Food Based on Fly Pre-Pupae

During BSF base-diet preparation, data showed two main types of consistencies. Pre-pupae developed in fruit waste had a final consistency of powder with a fat percentage of 29.37 ± 0.52% (F_3,11_ = 5031.57; *p <* 0.001; r^2^ = 99.95), larvae developed using restaurant and fish waste had a pasty consistency with a fat percentage of 46.54 ± 0.58 (F_3,11_ = 756.57; *p <* 0.001; r^2^ = 99.65) and 46.50 ± 0.45% (F_3,11_ = 30.00; *p <* 0.001; r^2^ = 91.84), respectively, while the control group registered 6.00 ± 0.25% (F_3,11_ = 2276.77; *p <* 0.001; r^2^ = 99.88). Regarding protein content, the pre-pupae developed using the restaurant and fish-waste substrates presented the same percentage as the control group (33.00 ± 0.50%). Significant differences in the data for moisture and ash content between all the treatments were observed (Table 4).

### 3.4. Development of Nile Tilapia

A significant increase in fish biomass was found for fish fed with the fish-waste-based diet (gain biomass = 5.88 ± 1.88 g) when compared to the control group (1.22 ± 1.10 g). This diet presented the highest weight gain when compared to the restaurant-waste-based diet (3.77 ± 2.15 g) and fruit-waste-based diet treatments (4.14 ± 1.67 g) (F_3,11_ = 10.54; *p <* 0.001; r^2^ = 49.70). Fish fed with the fish-waste-based diet reached a total length of 2.38 ± 0.71 cm, which was significantly higher than the rest of the treatments (0.62 ± 0.64 and 1.94 ± 0.66 cm) (F_3,11_ = 9.58; *p <* 0.001; r^2^ = 47.33). In addition, a greater gain in caudal length was observed for the fish-waste group (2.10 ± 0.56 cm), while the smallest values were observed in the control group (0.49 ± 0.50 cm) (F_3,11_ = 9.40; *p <* 0.001; r^2^ = 46.84) (Table 5).

### 3.5. Nutritional Analysis of Muscle Tissue from Nile Tilapia

Bromatological analysis showed that the fish-waste-based diet had the highest protein percentages (3.48 ± 0.03%) (F_3,11_ = 494.17; *p <* 0.001; r^2^ = 99.46), followed by the 100% commercial control group (3.06 ± 0.06%) (F_3,11_ = 8.63; *p =* 0.007; r^2^ = 76.40) and the fruit-waste-based diet (3.02 ± 0.20%) (F_3,11_ = 58.91; *p <* 0.001; r^2^ = 95.67). The restaurant-waste-based diet had the lowest protein content (2.23 ± 0.10%) (F_3,11_ = 4.75; *p =* 0.035; r^2^ = 64.05). The diet with the highest amount of crude fat was the fish-waste-based diet with 3.45 ± 0.03%, whereas we found the lowest fat content in the restaurant-waste-based diet (1.51 ± 0.10%) (Table 6).

## 4. Discussion

A diet based on insect-derived products is a protein alternative of interest for quality feeding [49]. Furthermore, insects are efficient biological conversion agents, their reproduction can be carried out at large scales, and they present a sustainable option [50].

### 4.1. Survival and Development of BSF Larvae

The present study determined the variation and growth and development responses of BSF larvae fed with food substrates based on organic waste. There are previous studies that have described the use of balanced food for developing BSF larvae. For example, Tomberlin et al. [39] evaluated larval development of BSFs with commercial food for laying birds and two commercial feeds, without finding significant differences in prepupal development or survival. Samayoa et al. [51] reported that the average larval development time for the BSF at 28 °C was 23.03 days, while the average development from egg to adult was 47.44 days. Conversely, results obtained in the present study showed a larvae development on the fruit-waste-based substrate of 22.87 days. Tomberlin et al. [39] reported that the BSF reared at 27 °C had an average development time egg–adult of 41.50 days (41–43 days) with a larval stage of 23.30 days (22.50–24.10 days). These data are quite similar to our findings, in which the restaurant-waste-based substrate recorded a time of 40.50 ± 0.50 d for adult-egg development, and the fruit-waste-based diet recorded a time of 22.87 days in the larval stage, at an average temperature of 27.00 ± 1.03 °C and relative humidity of 51.77 ± 6.23%. Variations found in our study could be attributed to the fact that the development time depends on several factors, such as the composition and proportion of waste from the food substrates, the environmental conditions of development, and the size of test populations [52].

Oonincx et al. [53] reported survival of 72.00 ± 12.90 to 86.00 ± 18.00% in diets planned with high and low amounts of protein. These findings are in agreement with our results, except for the case of commercial food. Barragan-Fonseca et al. [54] mentioned that larval survival of BSFs in meat waste including fish waste was 48.20 ± 8.70%. In contrast, in our experiment, larva survival in fish waste was twice as high (88.00 ± 1.15%). It is, however, important to mention that variations in the development time of the immature stages of insects may vary according to the space and number of individuals used in the rearing since there is competition for these factors.

### 4.2. Biomass of BSF Pre-Pupae and Length of Larvae

In our study, diet significantly affected larval growth, and this may be related to specific physicochemical characteristics of each substrate, which makes the insect–substrate interaction complex [55]. Insects can biodegrade organic wastes into insect biomass [56], which shows that substrate conversion efficiency implies a balance in food consumption to survive [57], indicating that insects can adapt to any quality of substrate [58]. Barroso et al. [59] and Surendra et al. [60] mentioned that the pre-pupae collected contained between 41 and 44% of proteins that can feed animals. However, the use of larvae or pre-pupae depends on their nutritional composition, as the levels of protein and fat absorption may vary according to the growth medium [61,62].

Nguyen et al. [37] reported that black soldier larval length reared on substrates based on kitchen waste was 20.80 mm, for fruit and vegetable waste it was 18.70 mm, and for the substrate based on fish waste it was 19.00 mm. This is similar to our findings, in which restaurant-waste-based substrate yielded lengths of 23.59 mm, for fruit-waste-based substrate it was19.00 mm, for fish-waste-based substrate, 19.89 mm, and for commercial food, 20.71 mm. Studies have shown that the type and amount of diet influence the growth (weight and height) of BSFs [63]. This is a key issue in the generation/development of adult or immature stages that allow the production of greater usable biomass [64,65], which can ensure a steady insect population [66].

Several studies have focused on the moisture content/development relationship (substrates with immature stages), and they have reported that a high moisture content increases larval growth, but decreases larval weight [67,68]. We observed this situation in the fruit-waste substrate, which presented the fastest development (31.33 + 0.47 d), but the lowest weight gain (0.17 + 0.04 g).

### 4.3. Bromatological Composition of Food Based on the BSF

St-Hilaire et al. [69] mentioned that the BSF larvae contain the fat omega-3 (3.0%), which makes it attractive to include in animal feed. In our study, the amount of protein was similar to commercial food in all treatments based on BSF pre-pupae (28–33%). However, Spranghers et al. [70] mentioned that their findings reached fat percentages between 39.9 and 43.1%, whereas Barragan-Fonseca et al. [54] reported a range between 37.0 and 63.0%. The percentage of ash found with the fruit-waste-based food was highest (13.88%) among the foods made with BSF pupae (6.32 and 9.00%), which was similar to the results found by Devic et al. [35]. Yet, the high ash content in the BSF-diet could be by deposition of calcium carbonate by the epidermis of the larval skin from the molting process [71]. In our study, moisture analysis was 12.0%, which was twice as high as in other studies (3.99 and 5.97%).

### 4.4. Development and Nutritional Analysis of the Nile Tilapia Muscle

We found significant differences in the growth and weight of *O. niloticus* when fed with integrated diets containing 70% of commercial food for tilapia and 30% of food based on fly pre-pupae. El-Saidy and Gaber [72] and Monentcham et al. [40] did not find significant variations in tilapia fed with 25 and 30% inclusion of BSF flours in fish feed until they increased the amount of feed to 1, 2, and 3% of weight daily, similar to the data recorded in our work. On the other hand, Sealey et al. [41] reported that diets including 25 and 50% of fly flour did not yield significant differences in the size and weight of fish, contrary to our finding.

Chatzifotis et al. [73] mentioned that the fat composition directly affected the proximal content of muscle in fish development. In our study, the fish-waste-based diet registered a 46.55% fat content and induced a greater gain in height and weight. Therefore, we recommend this level of fat for stable development of the fish, thus supporting the information cited by other authors mentioning that it is not convenient to completely replace the diet or make inclusions that exceed proportions of 40% of fly flour [42] unless partial defatting is carried out [9,54,74] to increase digestibility [75].

Cammack and Tomberlin [62] and Dzepe et al. [29] tested fruit waste and chicken-manure substrates with increasing moisture content (40 to 80%), observing that increasing the substrate moisture content increased larval feed, wet weight, development time, body size, and body thickness. Likewise, Dortmans et al. [76] commented that the ideal moisture content of food for BSF rearing was in the range of 70 to 80%, whereas, the lower threshold range was between 40 and 55%. Nguyen et al. [37] mentioned that larvae reared on substrates with high-fat content show accelerated development time since they more easily accumulate the necessary amount of fat to survive in the adult stage when they do not feed [77]. However, in our study, the fish-waste substrate had the second-lowest fat content, but the highest moisture and protein content, and had a faster development time compared to the other treatments.

Li et al. [78] mention that BSF larvae contain a high lipid content (26–35%), similar to that found in our study following biodegradation of substrates used for fly-larval rearing (29–46%), and state that this does not compromise fish development, which agrees with Ushakova et al. [79] who mentioned that BSF contain up to 45% of non-water-soluble compounds and recognize it as a biologically active natural resource. Lipid metabolism is an important development process of insects since it perform various functions in the organism such as energy storage, necessary for survival and reproduction in the adult stage [80], which are necessary factors when talking about the mass production of insects. Therefore, adding organic products with high-fat content in BSF-development substrates is an important factor for their production. However, the substrates to be used in BSF rearing, as well as the time of larval collection, must be carefully selected, since the fatty-acid composition of the substrate and the weight of the larvae affect fatty-acid composition in the larvae [81].

## 5. Conclusions

Results showed that an organic-waste mixture can produce high-quality BSF larvae that have the potential to substitute other sources of protein and lipids in commercial fish feed. The restaurant waste-based substrate extended the larval growth and the biological cycle of the BSFs, while the fish-waste-based substrate induced a lower egg–adult development period than the rest of the treatments. Pre-pupae reared on restaurant waste were higher in biomass, while the fruit-waste substrate produced a significantly lower weight. Regarding protein content, the pre-pupae developed using the restaurant and fish-waste substrates presented the same percentage as the commercial food. An increase in fish biomass was found for fish fed with the fish-waste-based diet. This diet presented the highest weight gain when compared to the restaurant and fruit-waste-based diet. Fish fed with a fish-waste-based diet reached a total length higher than the rest of the treatments. Bromatological analysis showed that the fish-waste-based diet had the highest crude fat and protein content. The nutritional composition of the food obtained from the fly pupae was similar in protein content to commercial food. Our findings indicate that rearing BSF pre-pupae on fish waste has the potential to deliver a high-quality insect resource with the potential for being incorporated into fish feed.

## Figures and Tables

**Table 1:** Survival (%) and development (days) of BSFs reared on diverse food-waste-based substrates.

Waste-Based Substrate	Hatching Time (days)	Development Days			Larval Survival (%)
		Larvae	Pupae	Total	
Restaurant waste	2.98 ± 0.21 a	20.95 ± 0.75 a	15.87 ± 0.85 a	40.50 ± 0.50 a	76.66 ± 3.33 a
Fruit waste	2.88 ± 0.22 a	22.87 ± 0.74 b	8.87 ± 0.75 c	34.87 ± 0.85 b	76.86 ± 12.13 a
Fish waste	2.99 ± 0.10 a	15.66 ± 0.57 c	12.93 ± 0.72 ab	31.33 ± 0.47 bc	88.00 ± 1.15 b
Commercial Food	2.98 ± 0.21 a	14.62 ± 0.47 cd	16.25 ± 0.50 a	33.00 ± 0.81 bc	98.88 ± 1.92 c

Means followed by the same lower-case letter within each column are not significantly different based on Tukey test at *p <* 0.05. Commercial food: tilapia food (Atilapia). Larval development is the average amount of time required to reach the prepupal stage. Pupal development is the amount of time that it remained in that state until the adult emerged. Total development is the number of days elapsed between when the eggs hatched and the adult emerged.

**Table 2:** Substrate chemical composition.

Sample	Moisture (%)	Ash (%)	Protein (%)	Fat (%)
Restaurant waste	71.87 ± 0.73 b	4.06 ± 015 c	14.5 ± 0.30 d	12.36 ± 0.37 a
Fruit waste	69.67 ± 0.51 c	2.46 ± 0.15 d	15.73 ± 0.32 c	2.73 ± 0.15 c
Fish waste	80.56 ± 0.50 a	5.60 ± 0.30 b	48.70 ± 0.43 a	5.90 ± 0.20 b
Commercial food (Atilapia^®^)	12.00 ± 0.10 d	10.03 ± 0.15 a	40.13 ± 0.32 b	5.96 ± 0.06 b

Means followed by the same lower-case letter within each column are not significantly different based on Tukey test at *p <* 0.05.

**Table 3:** Larval and pre-pupal length and weights of BSFs reared under different waste-based substrates.

Waste-Based Substrate	Larval wt (g)	Larval lt (mm)	Prepupal wt (g)	Prepupal lt (mm)
Restaurant waste	0.25 ± 0.04 a	23.59 ± 1.84 a	0.26 ± 0.05 a	23.78 ± 1.86 a
Fruit waste	0.17 ± 0.05 c	19.00 ± 3.33 b	0.17 ± 0.04 c	19.73 ± 1.75 b
Fish waste	0.21 ± 0.05 b	19.89 ± 2.58 b	0.21 ± 0.02 b	20.56 ± 1.62 b
Commercial Food	0.19 ± 0.05 bc	20.71 ± 2.25 b	0.26 ± 0.07 a	23.19 ± 2.61 a

wt, weight; lt, length. Means followed by the same lower-case letter within each column are not significantly different based on Tukey test at *p <* 0.05. Commercial food: tilapia food (Atilapia).

**Table 4:** Nutritional composition (%) of BSF pre-pupae meal fed with different organic waste.

Sample	Moisture (%)	Ash (%)	Fat (%)	Protein (%)
Restaurant waste	3.99 ± 0.01 ^d^	7.74 ± 0.01 ^c^	46.54 ± 0.58 ^a^	33.00 ± 1.00 ^a^
Fruit waste	5.97 ± 0.03 ^b^	13.88 ± 0.10 ^a^	29.37 ± 0.52 ^b^	28.00 ± 0.50 ^b^
Fish waste	5.05 ± 0.03 ^c^	6.32 ± 0.03 ^d^	46.50 ± 0.45 ^a^	33.00 ± 1.00 ^a^
Commercial food	12.00 ± 0.25 ^a^	9.00 ± 0.40 ^b^	6.00 ± 0.25 ^c^	33.00 ± 0.50 ^a^

Means followed by the same lower-case letter (a, b, c, d) within each column are not significantly different based on Tukey test at *p <* 0.05. Commercial food: tilapia food (Atilapia).

**Table 5:** Chronology of *O. niloticus* development fed on diets based on 30% of BSF meals.

Variable	Diets	Fish Sample						Total Gain (Biomass)
		1st Day	6th Day	12th Day	18th Day	24th Day	30th Day	
TL (cm)	dRW	5.54 ± 0.32 b	6.25 ± 0.30 a	6.41 ± 0.52 a	6.71 ± 0.47 b	6.90 ± 0.59 b	7.33 ± 0.57 b	1.79 ± 0.86 a
	dFW	5.73 ± 0.52 ab	6.21 ± 0.28 a	6.53 ± 0.38 ab	6.86 ± 0.48 ab	7.01 ± 0.42 b	7.67 ± 0.50 b	1.94 ± 0.66 a
	dFiW	5.81 ± 0.42 ab	6.43 ± 0.44 a	6.88 ± 0.60 ab	7.23 ± 0.39 a	7.67 ± 0.46 a	8.18 ± 0.53 a	2.38 ± 0.71 ab
	dHW	6.04 ± 0.26 a	6.33 ± 0.43 a	6.50 ± 0.32 b	6.64 ± 0.30 b	6.67 ± 0.40 b	6.67 ± 0.44 c	0.62 ± 0.64 c
CL (cm)	dRW	4.43 ± 0.48 a	5.07 ± 0.28 a	5.13 ± 0.47 a	5.38 ± 0.39 a	5.52 ± 0.48 b	5.89 ± 0.56 bc	1.44 ± 0.90 a
	dFW	4.54 ± 0.46 a	5.08 ± 0.28 a	5.31 ± 0.30 a	5.53 ± 0.48 a	5.64 ± 0.39 b	6.20 ± 0.45 ab	1.65 ± 0.61 a
	dFiW	4.58 ± 0.34 a	5.24 ± 0.40 a	5.62 ± 0.50 a	5.86 ± 0.38 a	6.24 ± 0.36 a	6.68 ± 0.44 a	2.10 ± 0.56 ab
	dHW	4.88 ± 0.25 a	5.18 ± 0.41 a	5.24 ± 0.25 a	5.47 ± 0.33 a	5.34 ± 0.32 b	5.38 ± 0.37 c	0.49 ± 0.50 c
W (g)	dRW	2.73 ± 0.51 b	4.17 ± 0.75 a	4.47 ± 1.23 a	4.61 ± 0.97 b	5.25 ± 1.28 b	6.51 ± 1.68 bc	3.77 ± 2.15 a
	dFW	3.40 ± 0.86 ab	4.31 ± 0.54 a	4.61 ± 0.82 a	5.51 ± 1.31 ab	5.87 ± 1.24 b	7.56 ± 1.33 ab	4.14 ± 1.67 a
	dFiW	3.23 ± 0.76 ab	4.97 ± 1.05 a	5.81 ± 1.59 a	6.18 ± 1.15 a	7.66 ± 1.51 a	9.11 ± 1.67 a	5.88 ± 1.88 ab
	dHW	3.76 ± 0.44 a	4.57 ± 1.14 a	4.65 ± 0.74 a	5.04 ± 0.85 ab	5.03 ± 0.92 b	4.98 ± 1.12 c	1.22 ± 1.10 c

Means (±SE) of three replicate aquaria (10 fish/aquarium). Means followed by the same lower-case letter within each column by variable (TL, CL, W) are not significantly different based on Tukey test at *p* < 0.05. TL, total length (cm); CL, caudal length (cm); W, weight (g); dRW (30% BSF from RW); dFW (30% BSF from FW); dFiW (30% BSF from FiW); dHW (100% commercial food for tilapia); RW, restaurant-waste-based substrate; FW, fruit-waste-based substrate; FiW, fish-waste-based substrate.

**Table 6:** Nutritional composition in the muscle of *O. niloticus* fed with diets based on 30% flour from the black soldier fly.

Sample	Moisture (%)	Ash (%)	Protein (%)	Fat (%)
dRW (30% BSF from RW)	76.85 ± 0.08 a	2.50 ± 0.05 ab	2.23 ± 0.10 c	1.51 ± 0.10 d
dFW (30% BSF from FW)	75.33 ± 0.50 b	2.42 ± 0.10 ab	3.02 ± 0.20 b	3.22 ± 0.10 b
dFiW (30% BSF from FiW)	75.12 ± 0.10 b	2.29 ± 0.10 b	3.48 ± 0.03 a	3.45 ± 0.03 a
dHW (100% commercial food for tilapia)	75.93 ± 0.75 ab	2.55 ± 0.10 a	3.06 ± 0.06 b	1.80 ± 0.05 c

Means followed by the same lower-case letter within each column are not significantly different based on Tukey test at *p <* 0.05. RW, restaurant-waste-based substrate; FW, fruit-waste-based substrate; FiW, fish-waste-based substrate.

## Data Availability

All the associated data are available in the manuscript.
